# Food Protein-Induced Enterocolitis Syndrome: Proposals for New Definitions

**DOI:** 10.3390/medicina55060216

**Published:** 2019-05-28

**Authors:** Stefano Miceli Sopo, Mariannita Gelsomino, Serena Rivetti, Ester Del Vescovo

**Affiliations:** Allergy Unit, Pediatrics Area, Department of Woman and Child Health, Policlinico Gemelli University Foundation IRCCS, Catholic University of Sacre Hearth, 00168 Rome, Italy; mariannita.gelsomino@gmail.com (M.G.); serena.rivetti@gmail.com (S.R.); esterdelvescovo@gmail.com (E.D.V.)

**Keywords:** Diagnostic criteria, food allergy, food protein-induced enterocolitis syndrome, oral food challenge, vomiting

## Abstract

Acute food protein-induced enterocolitis syndrome (FPIES) is a non-IgE-mediated allergy and is characterized by repetitive profuse vomiting episodes, often in association with pallor, lethargy, and diarrhea, presenting within 1–4 h from the ingestion of a triggering food. In 2017, the international consensus guidelines for the diagnosis and management of FPIES were published. They cover all aspects of this syndrome, which in recent decades has attracted the attention of pediatric allergists. In particular, the consensus proposed innovative diagnostic criteria. However, the diagnosis of acute FPIES is still currently discussed because the interest in this disease is relatively recent and, above all, there are no validated panels of diagnostic criteria. We propose some ideas for reflection on the diagnostic and suspicion criteria of acute FPIES with exemplary stories of children certainly or probably suffering from acute FPIES. For example, we believe that new definitions should be produced for mild forms of FPIES, multiple forms, and those with IgE-mediated symptoms. Moreover, we propose two clinical criteria to suspect acute FPIES and to refer the child to the diagnostic oral food challenge.

## 1. Introduction

Acute food protein-induced enterocolitis syndrome (FPIES) is a non-IgE-mediated allergy and is characterized by repetitive profuse vomiting episodes, often in association with pallor, lethargy, and diarrhea, presenting within 1–4 h from the ingestion of a triggering food. Cow’s milk, soy, grains, egg, and fish are among the commonest triggers in most countries, with regional variations. The pathogenetic mechanism underlying the syndrome remains poorly understood. It is suspected that the disorder is caused by the activation of intestinal T cells by food protein allergens, with the release of pro-inflammatory cytokines, resulting in an increased intestinal permeability and fluid passage into the lumen. The diagnosis of FPIES remains a clinical one. Oral food challenge (OFC) may be necessary to confirm the diagnosis in uncertain cases and in order to assess the disease resolution. The cornerstone of FPIES management is the avoidance of culprit food.

In 2017, the international consensus guidelines for the diagnosis and management of FPIES were published [1]. They certainly represent great progress because they have established important key points. Moreover, the international consensus proposed innovative diagnostic criteria, that are given in Table 1. However, the diagnosis of acute FPIES is still currently discussed [2], because the interest in this disease is relatively recent and, above all, there are no validated panels of diagnostic criteria. Prospective studies are even more necessary because “diagnosis of FPIES is based on clinical manifestations and requires a high index of suspicion, since we still lack a diagnostic laboratory tool” [3]. Unfortunately, we do not have standardized tools to code the high index of suspicion today. In fact, suspicion criteria are still lacking. Therefore, we will exemplify here some diagnostic uncertainties with the help of histories faced in our clinical practice. We hope it will be useful to start a collective reflection.

## 2. Cases Presentation

### 2.1. History No. 1

Livia, a 2-month-old baby, was admitted to hospital due to vomiting and lethargy. Two days before hospitalization, she had drunk 120 mL of formulated cow’s milk at 8:00 p.m. After two hours, she presented a single episode of vomiting without lethargy, pallor or diarrhea. The next day she drank 120 mL of formulated cow’s milk again and at the end of the feeding she had mild lethargy and sweating, but both disappeared within 15 min. She was taken to the emergency department, where she arrived in good clinical condition. During the physical examination, about two hours after the baby’s feeding, she presented a single episode of vomiting. Her blood tests (white blood cells count, pH) were normal. During the hospitalization, Livia was fine and she only drank breast-milk. She did not present other episodes of vomiting or lethargy. On the third day of hospitalization, an OFC was carried out with 100 mL of formulated cow’s milk. Livia presented one vomit, without lethargy or pallor, about 1 h and a half after the meal. In the following hours, the child did not show any further episodes of vomiting or any other signs or symptoms. The next day, the girl took extensively hydrolyzed cow’s milk without vomiting or other symptoms.

Comment: Livia presented, before the OFC, a single episode of vomiting about 2 h after drinking formulated cow’s milk in both cases. The timing and symptoms remind us of acute FPIES, but can we establish this diagnosis for Livia? According to the diagnostic criteria developed in 2017 [1] we cannot. Livia meets the major criterion, but she doesn’t meet ≥3 minor criteria. In fact, she had to go to the emergency department and she presented lethargy, which are only two minor criteria. Moreover, the lethargy has unusually preceded the vomiting by almost 2 h, it quickly resolved and it didn’t appear again during the OFC. Despite this, we believe that Livia’s history falls under the diagnosis of acute FPIES. In particular, we believe that it is similar to the mild form of acute FPIES. We can find the mild form of acute FPIES in table VI of the international consensus guidelines [1], which states that the mild form of acute FPIES can only consist of one vomit. We could establish the diagnosis of acute FPIES for this child if we referred to the Leonard et al. criteria [4], developed in 2012, which are defined as follows: (1) Less than 9 months of age at initial diagnosis; (2) repeated exposure to causative food elicits gastrointestinal symptoms without alternative cause; (3) absence of symptoms that may suggest an IgE-mediated reaction; (4) removal of causative food results in resolution of symptoms; (5) re-exposure or OFC elicits typical symptoms within 4 h. Livia’s history meets all five criteria of Leonard et al. [4], even before the OFC. We propose to include in the modern diagnostic criteria for FPIES details concerning the mild form. In fact, we believe to diagnose the mild form of acute FPIES, there should be at least three consecutive episodes with only one vomit, not accompanied by other signs or symptoms. Otherwise, we suggest to carry out the OFC, as we did with Livia.

### 2.2. History No. 2

Francesco has exclusively eaten formulated cow’s milk in his life. One day, when he was about three months old, he drank 120 mL of formulated cow’s milk at 6:00 a.m. At 8:00 a.m. he started vomiting. There were four episodes of vomiting and Francesco presented pallor and mild lethargy too. At 9:00 a.m. the baby felt good. The baby continued to drink the same formulated milk with no problems for 20 days. At 6:00 p.m. on the 21st day after the first episode of vomiting, Francesco drank 120 mL of the usual formulated cow’s milk. At 7:15 p.m. he started vomiting (four vomits) with severe pallor and lethargy.

He was taken to the emergency department, where his general condition was considered poor. Intravenous infusion of liquids has been started. His neutrophil counts were approximately 1.500/mmc and C-reactive protein was negative, and the abdominal ultrasound was normal. He did not have diarrhea. After 3 h from the onset of symptoms, Francesco was back to normal. He was hospitalized and during the following days, he was fine. All diagnostic tests were normal. He continued to be well, at least until his last follow-up visit three months after his hospital discharge.

Comment: Can Francesco receive a diagnosis of acute cow’s milk-induced FPIES only for his history and without OFC? Yes, he can according to the consensus criteria of 2017 [1]. Francesco meets the major criterion (vomiting after intake of cow’s milk and absence of IgE-mediated skin or respiratory reactions) and at least three minor criteria (a second episode of repetitive vomiting after consuming cow’s milk, the need for an emergency department visit, and the need for intravenous fluid support). However, no one would think to make this diagnosis for Francesco, because he drank formulated milk for 20 days with no problems between the first and the second episode of vomiting. In other panels of diagnostic criteria for acute FPIES [4,5,6,7,8], there is a criterion which states: “Removal of the offending protein from the diet resulted in resolution of the symptoms” and above all, that re-exposure to the culprit food again causes the symptoms. In the note to the consensus criteria [1] it is specified that: “The patient should be asymptomatic and growing normally when the offending food is eliminated from the diet.” But this specification does not necessarily imply that the patient should feel bad if the culprit food is not removed from the diet. Despite this, we believe it is necessary to specify this detail to diagnose acute FPIES. The consensus criteria of 2017 [1] do not expressly state that episodes of vomiting must be consecutive, and this may not be considered obvious. We propose to make it explicit, by including it in the necessary criteria.

### 2.3. History No. 3

Liam drank formulated cow’s milk (120 mL) for the first time in his life when he was three months old and he did not have any problems. When he was four months old, he drank formulated cow’s milk (120 mL) for the second time. After 3 h he drank breast milk and after another hour, he had three episodes of vomiting in rapid succession. He did not present pallor or lethargy. After two weeks, Liam drank formulated cow’s milk for the third time and he presented the same symptoms. The cow’s milk was eliminated from Liam’s diet. When the baby was five months old, he consumed two teaspoons of apple sauce for the first time in his life, and after 2 h and 30 min, he presented three vomits with mild pallor and mild lethargy. At the age of six months, Liam consumed six teaspoons of rice cream for the first time in his life and vomited after 3 h and 30 min (only one vomit, with no pallor or no lethargy). 

Comment: We cannot make the diagnosis of acute FPIES merely on the basis of Liam’s history, according to the criteria of 2017 [1]. In fact, Liam does not meet three minor criteria, he meets only two of them. Instead, according to other diagnostic criteria panels [4,5,6], Liam would receive the diagnosis. And after all, “if the history is clear with repeated episodes of delayed vomiting to the same or more than one identified food, the risks of OFCs may outweigh the benefits, and a presumed clinical diagnosis can be made without OFC”, state Leonard et al. [9] in an updated summary of the 2017 consensus [1]. Liam presented “repeated episodes of delayed vomiting to the same or more than one identified food”, so then can we make a diagnosis of acute FPIES for Liam or not? Moreover, can we say Liam is suffering from multiple acute FPIES? Can we say he is allergic to three food types? Should episodes be repeated for each food involved in multiple FPIES. What are the criteria to diagnose multiple acute FPIES? If acute FPIES has already been diagnosed for a food type, can the following episodes caused by other foods be judged with less rigor? Can even a single episode of vomiting be enough? Or should the criteria apply to each food be as strict as those applied to the first food type? We stand for less strict criteria, but we do not actually have specific diagnostic criteria for multiple acute FPIES.

### 2.4. History No. 4

During her life, Mia has had four episodes characterized by repeated vomiting, intense pallor, and lethargy. These episodes occurred 2 h and 30 min after the ingestion of fish and they regressed spontaneously in 2–3 h. One of these occurred at six months of life because of a homogenized sole, the second one at seven months of life because of 50 g of sole, the third one at eighteen months of life because of 100 g of flounder, and the last one at the age of two years because of 100 g of cod. A prick test with commercial cod extract was negative. Therefore, she meets the diagnostic criteria of the 2017 consensus [1]. When the baby was five years old, she performed an OFC with 100 g of sole for the verification of the possible acquisition of the tolerance. The test passed and Mia did not present any symptoms. After ten days, Mia ate 100 g of sole and she presented two episodes of vomiting after about 2 h and 30 min. She had no pallor or lethargy and after the vomit, she started playing again.

Comment: Is the last episode of vomiting—the one occurred ten days after the passed OFC with sole—attributable to a history of acute FPIES to fish? Mia’s mother refused another OFC. Even if the last episode was only a mild reaction, could it really have been a coincidence? If it was not a coincidence, how strong is the negative predictive value of the OFC? It is premature to doubt a 100% sensitivity during an OFC because we have not seen other cases like this and we have not read similar published stories. Regardless, when a parent—after a passed OFC—questions: From tomorrow can I give him the sole safely? Should we not consider Mia’s story?

### 2.5. History No. 5 

At the age of ten months, Rachele ate egg for the first time without having any adverse reaction. This consisted of one teaspoon of egg mixed with soup, which was boiled for at least 10 min. In the following two months, she ate an egg three more times (two teaspoons cooked in the soup for 10 min) and she always had repeated vomiting (five vomits each time) after 3–4 h from ingestion, along with pallor and lethargy. She did not show diarrhea and her symptoms regressed within about four hours. Her measurements of serum egg white-specific IgE was slightly positive (0.89 KU/L), so egg was eliminated from Rachele’s diet. At the age of two and a half years, Rachele was subjected to three OFC with a raw egg within five months, the prick-by-prick (PbP) test with raw egg was positive (the mean wheal diameter was 5 mm).

The OFCs were repeated because each time they were considered dubious and inconclusive. During the first one, the baby drank about 2/3 of a raw egg, mixed with cow’s milk and then refused to continue. After 15 min from the end of the ingestion, she presented a small vomit after an intense cry caused by the execution of an electrocardiogram, without other signs or symptoms for the next 4 h. The rub test with raw egg was negative. After two months, Rachele performed the second OFC. She ingested 1/4 of a raw egg mixed with cow’s milk and then refused to continue because of disgust. After twenty minutes from the end of the ingestion, she presented heavy vomiting and she complained of abdominal pain. She also presented mild erythema in the perioral region. After another two months, Rachele performed the third OFC with a raw egg inside the Italian dessert, tiramisu. The PbP test with raw egg was always positive (the mean wheal diameter was 6 mm). After twenty minutes, the child had eaten half a raw egg and then refused to continue. After 25 min from the end of the ingestion, she complained of abdominal pain. After another 15 min, she presented one vomit and after another 15 min, a second vomit. The rub test with raw egg was slightly positive. At the age of three years, Rachele performed the fourth OFC, this time with baked egg (without wheat matrix, an omelet in the oven at 180 °C for 30 min). The PbP test with baked egg was negative. The baby ate the whole omelet slowly, during the ingestion she complained of slight abdominal pain. She presented a vomit two hours after the beginning of the ingestion and one hour after its end.

Comment: Should all Rachele’s OFCs be considered positive? The fourth OFC allows a reflection on the positivity criteria of the OFC for acute FPIES (shown in Table 2). Outside the actual criteria, it is specified in a note [1] that: “The OFC will be considered diagnostic of FPIES (i.e., positive) if the major criterion is met with ≥2 minor criteria”. However, we suggest two important caveats to these criteria: (1) With the rapid use of ondansetron, many of the minor criteria, such as repetitive vomiting, pallor, and lethargy can be averted, and (2) not all facilities performing challenges have the ability to perform neutrophil counts in a timely manner. Therefore the treating physician might decide that a challenge be considered diagnostic in some instances, even if only the major criterion was met. However, in challenges performed for research purposes, providers should adhere to stringent criteria for challenge positivity.

In Rachele’s fourth OFC, the major criterion is respected, but none of the minor criteria are present. However, Leonard et al. [9] state: “If OFCs are performed in a controlled environment and symptoms are treated immediately, just the major criterion may be considered diagnostic”. Rachele’s symptoms have not been treated, so even in this case, may just the major criterion be considered diagnostic without the minor criteria? Could Rachel’s case be considered one of the instances where the treating physician might decide that a challenge be considered diagnostic, even if only the major criterion was met? How should we consider the vomiting that the child presented in the three previous OFCs performed with raw egg? All of them happened less than 1 h after the beginning of the egg ingestion. Furthermore, during the third OFC, the rub test was slightly positive. Rachele, like Livia, may have a mild form of acute FPIES and even her adverse reaction during OFC may be considered mild (just one vomit). In addition, it is likely that specific IgE plays a role in the pathogenesis of the child’s symptoms. We think that the positivity criteria of an OFC for FPIES must consider the mild form of acute FPIES, unlike what it is today, especially if the mild episodes are numerous: Rachel presented seven episodes between ingestions at home and during OFCs. Moreover, Rachel could be affected by a different, as well as a mild phenotype of acute FPIES, and this will be better clarified by the following history.

### 2.6. History No. 6

At the age of two years, Edoardo ate a small piece of walnut and presented cough, erythema, and swelling in the perioral region within 5 min. The prick test with commercial walnut extract was positive (a mean wheal diameter of 5 mm). At the age of three years, he performed an OFC with walnut. During the ingestion of the first doses of walnut, he presented mild perioral erythema. Then the child refused the second walnut dose and presented a vomit that wet his shirt. Where the vomiting wet shirt touched the skin, numerous wheals of hives appeared. The OFC was discontinued. After 1 h and 30 min from the interruption of the OFC, Edoardo presented a vomit with intense pallor and lethargy. The child vomited a second time after 15 min and he was suffering. After another 1 h, he was back to normal.

Comment: Can Edoardo’s failed OFC be judged positive according to the criteria of the International Consensus [1]? No, it cannot be, the major criterion involves the absence of classic IgE-mediated allergic skin or respiratory symptoms, the same is for other panels of diagnostic criteria [4,5,6,7,8]. In 2018, Leonard et al. [9] stated: “Awareness is increasing for FPIES, a non-IgE mediated food allergy characterized by delayed vomiting that typically presents in infancy.” However, is acute FPIES always and only non-IgE-mediated? We think there are some phenotypes of acute FPIES where serum IgE can play a pathogenic role and there are more indications in this direction [10]. The diagnostic criteria should include this possibility and remove the criterion of the absence of IgE-mediated signs or symptoms.

Consent for publication: All the names of the children mentioned are fictional. Informed consent was obtained from all individual participants included in the study.

## 3. Discussion

The six histories described here aim to highlight some points concerning the suspicion and diagnosis of acute FPIES. We would be happy to have new diagnostic definitions of phenotypes, according to the following.

- Mild FPIES: We propose to include in the modern diagnostic criteria for FPIES details concerning the mild form. In fact, we believe to diagnose the mild form of acute FPIES, there should be at least three consecutive episodes with only one vomit, not accompanied by other signs or symptoms. Otherwise, we suggest to carry out the OFC;
- Multiple FPIES: We propose that in the case of children who have already received a certain diagnosis of FPIES for one food, the possible diagnosis of FPIES for other foods should follow less stringent criteria. In our opinion, a single typical episode would suffice;
- FPIES with IgE-mediated symptoms: The diagnostic criteria should include this possibility and remove the criterion of the absence of IgE-mediated signs or symptoms.

Finally, we believe it is necessary that the consensus criteria of 2017 [1] explicitly state that episodes of vomiting must be consecutive.

Moreover, we would develop new definitions regarding the OFC positivity criteria for FPIES when:

- Symptoms are mild. We also think that the positivity criteria of OFC for FPIES must consider the mild form of acute FPIES, unlike what it is today, especially if the mild episodes are numerous.
- The symptoms are repeated at home after a passed OFC. Perhaps the sensitivity of the OFC for acute FPIES is not 100% and we should warn parents about this.

Certainly, the international consensus criteria of 2017 [1] constituted an important moment: They were the result of numerous expert opinions and they proposed an innovative way to diagnose acute FPIES. However, we believe this important diagnostic aspect still needs to be perfected, in anticipation of a prospective study that allows the development of stronger criteria. The lack of this kind of study is a serious issue.

Moreover, there are no published criteria of suspicion, on the basis of which to refer a child with suspected acute FPIES to the OFC. Of course, no one would suspect the FPIES for a child who does not want to eat a certain food, who cries intensely and vomits 5 min after a first bite. And no one would suspect FPIES when between two compatible episodes, there are repeated harmless ingestions of the suspected food, as in Francesco’s story. But if a child vomits repeatedly 2 h after food ingestion, should we consider the diagnosis of FPIES, eliminate that food from the child’s diet and propose an OFC? We do not think so. And if he also shows lethargy and pallor, what should we do? In short, standardized tools in processing suspicion are necessary. Did Livia have a history such as to induce suspicion? May we suspect FPIES if there are two consecutive episodes with only one vomit at 2 h from the ingestion of a certain food, even without considering the unusual lethargy that preceded the second episode of vomiting? Yes, we can, in our opinion. We propose some suspicion criteria in Table 3. Perhaps some readers will want to add more.

## Figures and Tables

**Table 1:** Diagnostic criteria for patients presenting with possible acute food protein-induced enterocolitis syndrome FPIES (from reference [1]).

**Major criterion**
• Vomiting in the 1–4 h period after ingestion of the suspect food and the absence of classic IgE-mediated allergic skin or respiratory symptoms.
**Minor criteria**
A second (or more) episode of repetitive vomiting after eating the same suspect food; Repetitive vomiting episode 1–4 h after eating a different food; Extreme lethargy with any suspected reaction; Marked pallor with any suspected reaction; The need for an emergency room visit with any suspected reaction; The need for intravenous fluid support with any suspected reaction; Diarrhea in 24 h (usually 5–10 h); Hypotension; Hypothermia.

The diagnosis of FPIES requires that a patient meets the major criterion and ≥3 minor criteria. If only a single episode has occurred, a diagnostic OFC should be strongly considered to confirm the diagnosis, especially because viral gastroenteritis is so common in this age group. Furthermore, although not a criteria for diagnosis, it is important to recognize that acute FPIES reactions will typically completely resolve over a matter of hours compared with the usual several-day time course of gastroenteritis. The patient should be asymptomatic and growing normally when the offending food is eliminated from the diet.

**Table 2:** Diagnostic criteria for the interpretation of oral food challenges (OFCs) in patients with a history of possible or confirmed FPIES (from reference [1]).

**Major criterion**
• Vomiting in the 1 to 4 h period after ingestion of the suspect food and the absence of classic IgE-mediated allergic skin or respiratory symptoms.
**Minor criteria**
Lethargy; Pallor; Diarrhea 5–10 h after food ingestion; Hypotension; Hypothermia; An increased neutrophil count of >1500 neutrophils above the baseline count.

The OFC will be considered diagnostic of FPIES (i.e., positive) if the major criterion is met with ≥2 minor criteria. However, we would suggest two important caveats to these criteria: (1) With the rapid use of ondansetron, many of the minor criteria, such as repetitive vomiting, pallor, and lethargy can be averted, and (2) not all facilities performing challenges have the ability to perform neutrophil counts in a timely manner. Therefore, the treating physician might decide that a challenge be considered diagnostic in some instances, even if only the major criterion was met. However, in challenges performed for research purposes, providers should adhere to stringent criteria for challenge positivity.

**Table 3:** Suspicion criteria for acute FPIES.

Suspicion criteria for acute FPIES.
• A single episode of projectile and repeated vomiting, occurring 2–4 h after food intake, accompanied by at least moderate pallor and lethargy. There should not be ingestion of the same food without adverse reactions after the episode of vomiting.
• At least two consecutive episodes of one (not repetitive) vomit, without pallor and lethargy, within 2–4 h from ingestion of the same food. There should not be ingestion of the same food without adverse reactions after episodes of vomiting.
